# Advances in the antitumor mechanisms of tripartite motif-containing protein 3

**DOI:** 10.1007/s00432-024-05632-6

**Published:** 2024-02-27

**Authors:** Wei Teng, Yuanguo Ling, Zongwei Liu, Lishi Jiang, Genyuan Fu, Xingwang Zhou, Niya Long, Jian Liu, Liangzhao Chu

**Affiliations:** 1 Department of Neurosurgery, The Affiliated Hospital of Guizhou Medical University, Guiyang, Guizhou Province People’s Republic of China; 2 Department of Clinical Medicine, Guizhou Medical University, No. 9 Beijing Road, Guiyang, Guizhou China; 3 Department of Neurosurgery, Guizhou Provincial People’s Hospital, Guiyang, Guizhou Province People’s Republic of China

**Keywords:** TRIM3, Biological function, Antitumor mechanism, E3 ligase

## Abstract

The tripartite motif-containing (TRIM) protein family has steadily become a hotspot in tumor-related research. As a member of the E3 ubiquitin ligase family, TRIM is working on many crucial biological processes, including the regulation of tumor cell proliferation, metastasis, apoptosis, and autophagy. Among the diverse TRIM superfamily members, TRIM3 operates via different mechanisms in various types of tumors. This review primarily focuses on the current state of research regarding the antitumor mechanisms of TRIM3 in different cancers. A more in-depth study of TRIM3 may provide new directions for future antitumor treatments. Our review focuses on TRIM3 proteins and cancer. We searched for relevant articles on the mechanisms by which TRIM3 affects tumorigenesis and development from 1997 to 2023 and summarized the latest progress and future directions. Triad-containing motif protein 3 (TRIM3) is an important protein, which plays a key role in the process of tumorigenesis and development. The comprehensive exploration of TRIM3 is anticipated to pave the way for future advancements in antitumor therapy, which is expected to be a new hallmark for cancer detection and a novel target for drug action. TRIM3 is poised to become a significant milestone in cancer detection and a promising focal point for drug intervention. Recent years have witnessed notable progress in research aimed at unraveling the antitumor mechanism of TRIM3, with far-reaching implications for practical tumor diagnosis, treatment protocols, efficacy evaluation, economics, and pharmaceutical utilization.

## Introduction

The tripartite motif-containing (TRIM) protein family, a group of highly conserved E3 ubiquitin ligase proteins, plays indispensable roles in the initiation, progression, and resistance of various cancers (Hatakeyama 2011). These proteins exhibit bifunctionality, acting as either cancer promoters or tumor suppressors, the function of which is contingent on the specific cancer type (Connacher and Goldstrohm 2021). Furthermore, it turns out that the TRIM family could regulate copious biological processes. These include, but are not limited to, epithelial–mesenchymal transition (EMT), the adoption of cancer stem cell (CSC) phenotypes (Wang et al. 2022), cell cycle modulation, DNA repair, stress response, apoptosis, and autophagy (Crawford et al. 2018).

TRIM family plays a crucial role in human cancers. TRIM3 is an important member of this family, which has been under the spotlight of cancer research in recent years. Investigations have shown that the human TRIM3 gene resides in the chromosomal region 11p15.5. Intriguingly, the deletion of this region is correlated with various cancer types, hinting at a potential tumor-suppressive role of TRIM3 (Ozato et al. 2008). This review provides a comprehensive examination of the physiological function of TRIM3, focusing on its participation in antitumorigenesis and cancer development. This information should serve as a valuable foundation for future studies on TRIM3.

## The structure and biological function of TRIM3

**Fig. 1:**
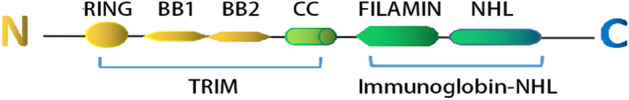
Diagram of TRIM-NHL functional domain

**Fig. 2:**
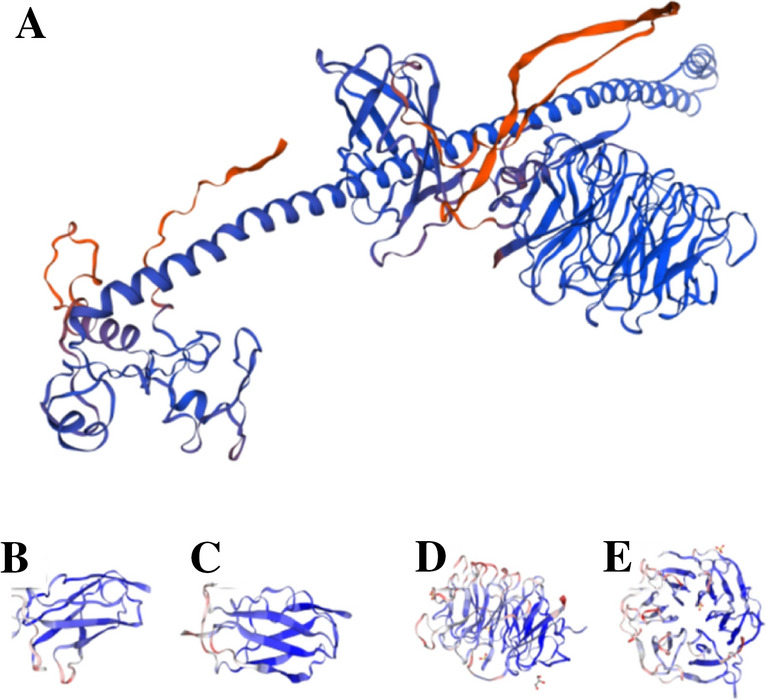
TRIM3 protein structural domain pattern diagram. TRIM3 protein structural domain pattern diagram. **A** TRIM3 protein pattern diagram; **B** Filamin structural domain longitudinal schematic; **C** Filamin structural domain transverse schematic; **D** NHL structural domain longitudinal schematic; **E** NHL structural domain transverse schematic. Photograph credit: α Folded Protein Structure Database (ebi.ac.uk)

**Fig. 3:**
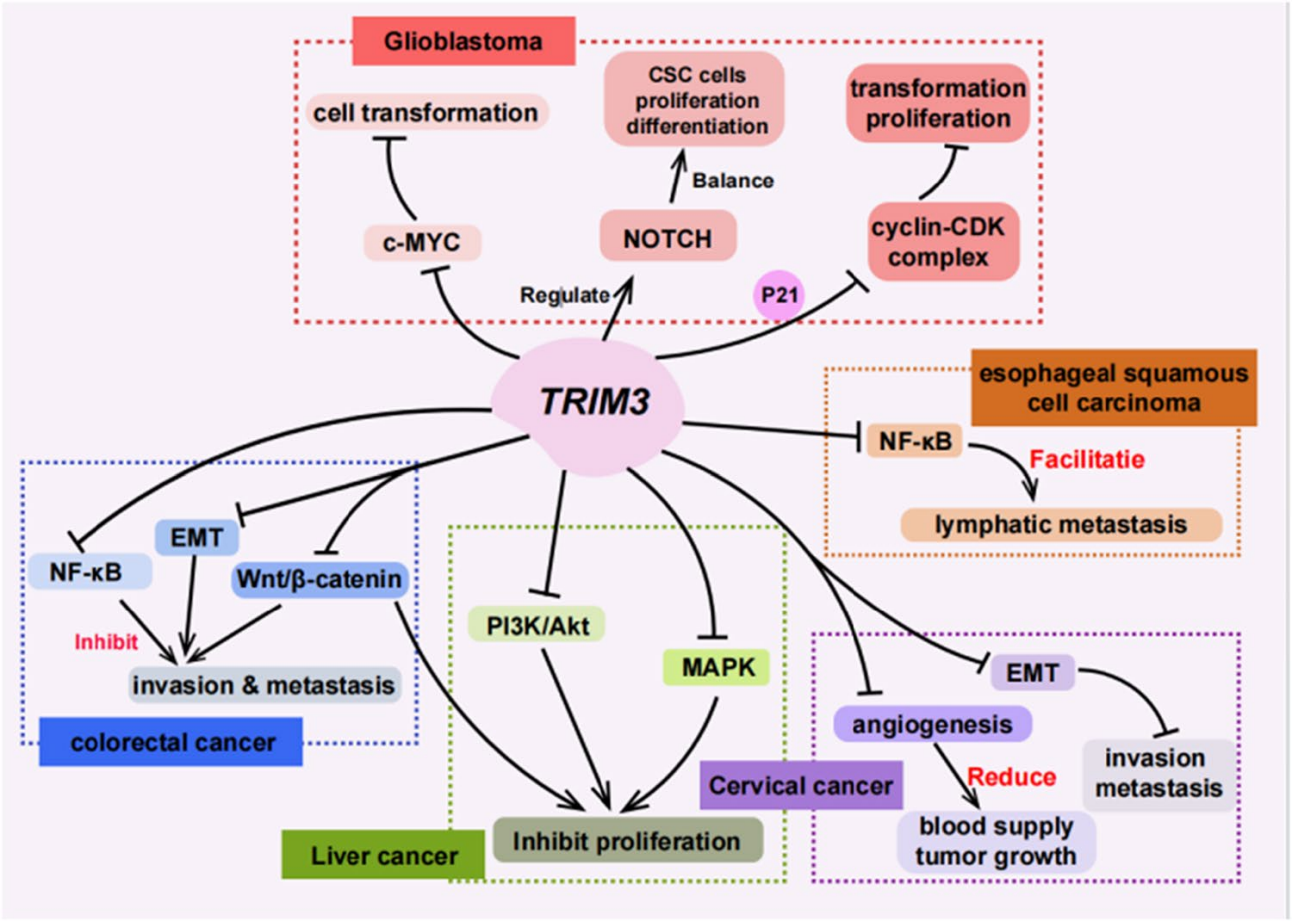
Mechanism of playing a tumor suppressor role via TRIM3 protein

The TRIM3 protein could be portrayed through three primary domains: a RING finger domain, one or two B-BOX type zinc finger (BB1 and BB2) structures, and a coiled-coil (CC) structure (Figs. 1 and 2) (Reymond et al. 2001). The RING domain comprises a consistent arrangement of cysteine (Cys-C) and histidine (His-H) residues, which interact synergically with zinc ions in a cross-brace configuration (Fig. 3). Specifically, Cys residues at sites 1, 2, 5, and 6 bind to the first zinc ion, while His residues at sites 3, 4, 7, and 8 bind to the second zinc ion (Freemont 2000). The RING domain of TRIM3 falls within the single-subunit classification of E3 ubiquitin-protein ligases, bestowing TRIM3 with E3 ubiquitin ligase activity. The B-BOX domain, encompassing BB1 and BB2, is organized from the N-terminus to the C-terminus. Each B-BOX can adopt one of two distinct zinc-binding motifs. The consensus sequence for B-BOX type 1 (B-BOX1) is C-X(2)-C-X(7–10)-C-X(2)-C-X(4–5)-C-X(2)-C(H)-X(3–6)H-X(2–8)-H(C5(C/H)H2), whereas for B-BOX type 2 (B-BOX2), it is C-X(2)-4-C/H-X(7)10-C-X(7)-C-X(2)-C-X(3)-6-H-X(2)-8-H(C(C/H)C3H2). The existence of B-BOX2 is a distinguishing feature of RBCC (TRIM) proteins. While the specific work of the CC domain has yet to be fully clarified, it is known to be embroiled in mRNA regulation (Roshanazadeh et al. 2022). The NHL domain, positioned at the C-terminus of the TRIM3 protein, derives its name from NCL-1/HT2A/LIN-41, the original protein designation. Typically, an immunoglobulin-like domain precedes the NHL domain. Typically, an immunoglobulin-like domain precedes the NHL domain (Deyne et al. 1998).

TRIM3, one kind of E3 ubiquitin ligases, is widely distributed within several subcellular organelles including the cell nucleus, cytosol, and endoplasmic reticulum and is expressed in a variety of cell types. It possesses both transcriptional repression activity and E3 ligase activity (Shen et al. 1997, 1998). Importantly, TRIM3 exerts substantial influence over multiple essential physiological processes within an organism, including cell proliferation, DNA damage repair, intracellular signaling, and immune response (Lau et al. 2010). By enhancing the activity of caspase 3, it induces apoptosis in normal cell processes. It also intervenes in the NF-κB signaling pathway by ubiquitinating and degrading NF-κB inhibitor IκBα, thereby exercising critical regulatory functions in innate immunity (Weatheritt et al. 2014). Furthermore, TRIM3’s binding affinity with p21 WAF1/CIP1 impedes its nuclear accumulation, effectively inhibiting tumor growth by impeding the promotion of cyclin D1-CDK4 in the cell cycle (Wulczyn et al. 2007). Recent studies have further illuminated TRIM3’s involvement in key signaling pathways during cancer progression. For example, it modulates the Notch signaling pathway, the P38 signaling pathway, and mechanisms related to cell cycle, tumor cell stemness, and EMT (Zuo et al. 2023; Dai et al. 2021).

## The role of TRIM3 in antitumorigenesis and tumor development.

### The antitumor effect of TRIM3 on glioblastoma

Glioblastoma multiforme (GBM) is an aggressive primary brain tumor and ranks among the deadliest central nervous system (CNS) tumors in adults. Despite significant advancements in clinical treatments, such as surgical interventions, temozolomide drug therapy, and gamma-ray therapy, the long-term survival rate for patients is only around 5% after five years (Stupp et al. 2005; Ostrom et al. 2018; Wen and Kesari 2008). Research suggests that the loss of heterozygosity on chromosome segment 11p15.5 in malignant gliomas may indicate TRIM3 as a potential candidate tumor suppressor gene for brain tumors (Tian et al. 2022).

#### Inhibition on GBM cells in vitro and in vivo

The growth of GBM cells in vitro and in vivo can be influenced by various factors, including environmental and nutritional factors and the regulation of certain tumor suppressor genes. Among these, TRIM3, a prototypical E3 ubiquitin ligase, assumes a vital role in glioblastoma. In experiments utilizing orthotopic tumor-bearing nude mice, it was viewed that mice engrafted with U87-TRIM3 cells exhibited a longer median survival time, indicating that TRIM3 expression exerts an inhibitory effect on glioblastoma in vivo. Moreover, GBM cells overexpressing TRIM3 demonstrated a marked reduction in nonadherent colony formation in soft agar during a 30-day period, providing strong evidence that TRIM3 expression stifles the proliferation and colony formation of GBM cells in vitro (Chen et al. 2014).

#### Inhibiting the expression and activity of c-myc and affecting cell transformation

The c-myc gene is an important constituent of the myc gene family which has a prominent function in cell apoptosis and has been implicated in the development of various tumors (Dang 1999). Analysis of GBM data from TCGA revealed a pattern of c-myc overexpression and TRIM3 underexpression in nearly all GBM samples. Fruit fly studies have illustrated that the Brat protein partially restrains c-myc, facilitating asymmetric cell division and neural differentiation (Lee et al. 2006^).^ In cultured glioblastoma cell lines, TRIM3 has been noticed to inhibit the levels and transcriptional activity of c-myc. Evidence points to a marked negative correlation between TRIM3 expression and oncogene expression within the same sample. They demonstrated TRIM3's inhibitory effect on c-myc transcriptional activity and expression levels in glioblastoma by employing the c-myc-driven luciferase reporter gene in U87 MG and LN 229 GBM cell lines (Chen et al. 2014).

#### Inhibiting the cyclin-CDK complex by binding to P21 and affecting the proliferation

P21 is a pivotal player within the cyclin-dependent kinase (CDK) inhibitor family and is intimately associated with tumor suppressor functions. It orchestrates cell cycle progression, DNA replication, and repair by reducing the activity of CDK complexes, thereby establishing a tight nexus between tumor suppression and cell cycle control processes (Abbas and Dutta 2009). Recent studies have shown that in the RCASPDGF-HA/nestin-TvA model, p21 acts as an assembly factor for cyclin D-CDK4, and TRIM3 can bind to p21 to inhibit cell proliferation. The underlying mechanism may involve TRIM3 and the cell cycle protein-CDK complex competing interaction with p21 (Mukherjee et al. 2016).

#### Regulating NOTCH signaling to balance the stem cell proliferation and differentiation

The receptors encoded by NOTCH genes are members of a highly conserved class of cell surface receptors that regulate the development of various biological cells, ranging from sea urchins to humans. NOTCH signaling influences multiple processes in normal cell morphogenesis, such as the differentiation of pluripotent progenitor cells, cell apoptosis, proliferation, and the formation of cell boundaries. The myriad phenotypic alterations resulting from mutations in NOTCH gene loci underscore the diversity of NOTCH signaling effects (Bray 2006). NOTCH has a strong regulatory effect on GBM stem cell behavior (Battiste et al. 2007). As an RNA-binding protein, Musashi has the function of triggering NOTCH signaling by hindering Numb translation and averting the degradation of NOTCH-1. Typically, Numb affixes to the intracellular domain of NOTCH-1 (NICD) and lowers the expression of downstream targets, e.g., Hes-1. In NHNP-controlled neural spheres (NHNP-GFP) studied by Subhas Mukherjee et al., high basal TRIM3 expression was found to be associated with low Musashi expression, high Numb expression, and low Notch activity. In NHNP neural spheres (NHNP-sh-TRIM3), inhibiting TRIM3 caused an upregulation of Musashi, downregulation of Numb, and elevated expression of the NOTCH-1 target Hes-1. Mirroring these results in NHNP, the repair of TRIM3 in GBM neural spheres resulted in a decrease in Musashi and Hes-1 levels, thus establishing an inverse relationship between TRIM 3 and Musashi expression (Ji et al. 2021). Therefore, TRIM3 is crucial in regulating the Musashi–Numb–Notch signaling.

### The antitumor effect of TRIM3 on liver cancer

Liver cancer is a prevalent malignancy with escalating incidence and mortality rate globally. Addressing those challenges and limitations in liver cancer treatment is essential to enhance patient survival. Emerging research has demonstrated that TRIM3 expression is diminished in liver cancer and exhibits pronounced anticancer effects (Huang et al. 2017). Therefore, exploring TRIM3 as a potential therapeutic target could pave the way for novel strategies to combat liver cancer and improve patient outcomes.

#### Inhibition of hepatoma cell proliferation

Overexpression of TRIM3 can curtail the proliferation capacity of liver cancer cells by regulating related signaling pathways, such as PI3K/Akt, MAPK, and Wnt/β-catenin, which affect the cell cycle and proliferation-related gene expression (Mukherjee et al. 2016). Additionally, the enhanced expression of TRIM3 can suppress their migration and invasive ability by blocking EMT and expression of metastasis-related genes, such as E-cadherin, N-cadherin, and vimentin (Lu et al. 2022). In addition, TRIM3 overexpression reduces the number of tumor stem cells and hinders tumor angiogenesis, effectively restraining tumor growth and metastasis in liver cancer.

#### Inducing Go/G phase arrest and affecting cell cycle progression

TRIM3 mitigates tumor growth in liver cancer by making cell cycle arrest in tumor cells. Besides liver cancer cells with low TRIM3 expression, those with high TRIM3 expression exhibit a noteworthy boost in the ratio of cells in the Go/G1 phase and a significant decrease in the percentage of cells in the G2/M phase. Conversely, when the expression of TRIM3 is reduced, the proportion of liver cancer cells in the S and G2/M phases increases. These finding clearly indicates that TRIM3 can induce Go-/G1-phase arrest in liver cancer cells, thereby influencing the cell cycle progression and inhibiting tumor cell proliferation. Hence, TRIM3 has a certain inhibitory job in the occurrence and development of liver cancer (Huang et al. 2017).

### Inhibitory effect of TRIM3 on colorectal cancer

Colorectal cancer, a malignancy deriving from the epithelial cells of the colon or rectum, is a common digestive system tumor predominantly affecting individuals aged 50 and above, with incidence rates increasing with age (Kahi et al. 2016). Recent studies showed that the expression level of TRIM3 in colorectal cancer is much less than that in normal colorectal tissue (Hiltunen et al. 2022). A particular study identified a significant downregulation of TRIM3 in colorectal cancer tissues, and the expression level of TRIM3 was closely associated with the clinical and pathological hallmarks of colorectal cancer. Furthermore, the expression level of TRIM3 has a direct link with the prognosis of colorectal cancer, as sufferers with lesser TRIM3 expression tend to experience poorer outcomes (Betschinger and Knoblich 2004).

#### Inhibiting the proliferation

The overexpression of TRIM3 significantly curbs the proliferation and growth, while simultaneously resulting in apoptosis of colorectal cancer cells (Han et al. 2023). The Wnt/β-catenin signaling pathway has a critical influence on the occurrence and growth of colorectal cancer. Studies have shown that TRIM3 can block the activation of the Wnt/ beta-catenin signaling pathway, thereby hindering cell proliferation and invasion. By reducing the stability of β-catenin, TRIM3 effectively obstructs the activation of the Wnt/β-catenin signaling pathway (Hu and Gan 2017). Furthermore, TRIM3 exerts its inhibitory action by suppressing the expression of downstream target genes in the Wnt/β-catenin signaling pathway, such as c-Myc and CyclinD1, thus curtailing the proliferation and invasion of colorectal cancer cells (Song et al. 2019). Consequently, TRIM3 also limits the proliferation and self-renewing capabilities of colorectal cancer stem cells, thus inhibiting the onset and development of colorectal cancer.

#### Inhibiting the invasion and metastasis

TRIM3 demonstrates significant potential in inhibiting the invasion and metastasis of colorectal cancer cells. A study has revealed that overexpression of TRIM3 leads to a substantial diminution in the invasion and metastasis capabilities of colorectal cancer cells, along with a decreased migration ability. Additionally, TRIM3 effectively curbs the colorectal EMT process, hindering their metastatic and invasive properties (Song et al. 2018). Furthermore, TRIM3 induces apoptosis in colorectal cancer cells and regulates their metabolism, effectively impeding the occurrence and progression of colorectal cancer. The NF-κB signaling pathway also holds importance in the development and advancement of colorectal cancer (Boulay et al. 2009). Research has demonstrated that TRIM3 acts as a hindrance to the NF-κB signaling pathway, creating a decreased proliferation and invasion of colorectal cancer cells. By hindering the nuclear transport and DNA-binding capacity of NF-κB, TRIM3 effectively deactivates this signaling pathway and downregulates the downstream target genes, such as IL-6 and TNF-α, thus curbing the inflammatory response and invasion abilities of colorectal cancer cells (Zhu et al. 2023). As a result, TRIM3 emerges as a promising new target for the treatment of colorectal cancer, given its potential to inhibit invasion and metastasis, induce apoptosis, and regulate essential signaling pathways involved in colorectal cancer progression.

#### Regulation of cellular metabolism

TRIM3 can regulate the metabolism of colorectal cancer cells. One study found that overexpression of TRIM3 can inhibit metabolic pathways such as glycolysis and oxidative phosphorylation in colorectal cancer cells, causing the quelling of their growth and invasion (Xie et al. 2020). TRIM3 can also modulate the mitochondrial function of colorectal cancer cells, further contributing to the impediment of their growth and invasion.

#### Regulation of PI3K/Akt signaling pathway

The PI3K/Akt signaling pathway also possesses an extremely important mission in the happening and development of colorectal cancer. Research has shown that TRIM3 can hinder the energizing of the PI3K/Akt signaling, effectively restraining the proliferation and invasion of colorectal cancer cells (Eberhardt et al. 2020). By reducing the phosphorylation level of Akt, TRIM3 effectively hampers the activation of the PI3K/Akt signaling pathway. In addition, TRIM3 can also suppress the expression of downstream target genes in the PI3K/Akt signaling pathway, such as Bcl-2 and Mcl-1, thus promoting apoptosis in colorectal cancer cells (Wang et al. 2020).

### Tumor-suppressive effect of TRIM3 in cervical cancer

#### Inhibiting the proliferation

TRIM3 overexpression has a substantial inhibitory effect on the proliferation capacity of cervical cancer cells. Research has found that TRIM3 regulates multiple signaling pathways and related molecules to suppress cell proliferation (Jiang et al. 2022). For instance, TRIM3 downregulates the activity of the PI3K/Akt and MAPK signaling pathways, causing the inhibition of cell proliferation and proliferation-related gene expression. Additionally, TRIM3 overexpression also inhibits the activity of the Wnt/β-catenin signaling pathway, thereby influencing cell proliferation and transcriptional regulation (Chen et al. 2017).

#### Inhibiting the migration and invasion

TRIM3 overexpression significantly hinders the migration and invasion capabilities of cervical cancer cells. This inhibition is accomplished through the regulation of transcription factors and mediation of signaling molecules crucial for the EMT process, which is essential for cell migration and invasion. Notably, TRIM3 downregulates the expression of EMT transcription factors, such as Snail, Slug, and TWIST, thus curbing the EMT process in cervical cancer cells and weakening their migration and invasion potentials (Diao et al. 2020). Furthermore, TRIM3 overexpression exhibits tumor-inhibiting effects in cervical cancer. Research reveals that TRIM3 reduces the population of cervical cancer tumor stem cells and actively participates in regulating their apoptosis and differentiation by engaging various signaling pathways (Diao et al. 2020). Moreover, TRIM3 overexpression effectively suppresses angiogenesis, resulting in decreased tumor blood supply and growth. These findings underscore the potential of TRIM3 as a valuable target for therapeutic interventions in cervical cancer treatment.

### Function of TRIM3 in esophageal squamous cell carcinoma

In the current research, it has been demonstrated that TRIM3 coacts with the E4 ligase-dependent proteasomal turnover of importin α3 and α-actinin-4 (ACTN4), which successively modulates the nuclear factor κB (NF-κB) at a stable level(Zhu et al. 2019 Apr). Heterozygous deletion-mediated downregulation of TRIM3 disrupts the NF-κB-IκB-α negative-feedback loop, thereby promoting symmetric dimethylation of NF-κB/p65 at Arg65 and Arg65. This enhances p65 DNA-binding affinity and transcriptional activity, consequently facilitating lymphatic metastasis of esophageal squamous cell carcinoma (ESCC) cells and triggering the activation of NF-κB signaling(Zhao et al. 2022 Sep).

### Role of TRIM3 in lung cancer

Lung cancer stands as one of the most prevalent and deadliest malignancies worldwide. Recent investigations have highlighted the crucial role of TRIM3 in suppressing lung cancer (Zhan et al. 2015). A study demonstrated significantly lower expression levels of TRIM3 in lung cancer tissue compared to normal lung tissue, with its expression level closely correlating with the clinical and pathological characteristics of lung cancer. Further experiments evidenced TRIM3’s ability to inhibit lung cancer cell proliferation and invasion and induce apoptosis (Altinoz et al. 2016). Additionally, TRIM3 can also regulate the metabolism of lung cancer cells, curtailing metabolic pathways such as glycolysis and oxidative phosphorylation, thus hindering the growth and invasion of lung cancer cells. The anticancer effect of TRIM3 is mainly achieved by regulating the NF-κB signaling pathway (Choi et al. 2014). TRIM3 can diminish the activation of NF-κB, thereby suppressing the proliferation and invasion of lung cancer cells.

### Summary of TRIM3 antitumor mechanisms in different tumors

Abnormal expression of TRIM3 is closely linked to the occurrence and progression of various tumors, including glioma, liver cancer, and colorectal cancer (Table 1). When TRIM3 is overexpressed in these tumors, it exhibits a promising anticancer effect, and it is mainly not only through the regulation of the NF-κB signaling pathway, Notch signaling pathway, and P38 signaling pathway, but also through the regulation of cell cycle, regulation of tumor cell dryness, EMT, and other mechanisms to play a tumor inhibitory role, although the specific biological behaviors and underlying mechanisms may vary among different tumor types. At present, there are few studies on this gene protein, and the antitumor mechanism of TRIM3 is still not completely clear. Its molecular mechanism and signaling pathway will be further explored in future. Therefore, further comprehensive and in-depth multi-omics studies are warranted to elucidate the specific mechanisms through which TRIM3 operates in these varied tumors.

**Table 1:** Summary of TRIM3 antitumor mechanisms in different tumors

Cancer type	Mechanisms	References
Glioma	Inhibiting c-myc genes and cyclin-CDK complexes (P21 conjunction) for affecting GBM cell transformation and proliferation, regulating NOTCH signaling transduction, and balancing stem cell proliferation and differentiation	Chen et al. 2014; Dang 1999; Lee et al. 2006; Abbas and Dutta 2009; Mukherjee et al. 2016; Bray 2006; Battiste et al. 2007; Ji et al. 2021)
Liver cancer	Regulating signaling pathways such as PI3K/Akt, MAPK, and Wnt/β-catenin, affecting the expression of cell cycle and proliferation-related genes, inducing cell cycle arrest at the G0/G1 phase, and reducing the proliferation	Mukherjee et al. 2016) (Huang et al. 2017; Lu et al. 2022)
colorectal cancer	Inhibiting Wnt/β-catenin and NF-κB signaling pathways and downstream target genes and suppressing the EMT process for proliferation and invasion inhibition and cellular inflammatory responses reduction	Kahi et al. 2016; Hiltunen et al. 2022 Mar; Betschinger and Knoblich 2004; Han et al. 2023; Hu and Gan 2017; Song et al. 2019; Song et al. 2018; Boulay et al. 2009; Zhu et al. 2023; Xie et al. 2020; Eberhardt et al. 2020; Wang et al. 2020)
Cervical cancer	Downregulating the PI3K/Akt, MAPK, and Wnt/ β-catenin signaling pathways for cell proliferation inhibition; inhibiting the EMT process, thus weakening migration and invasive capabilities; and suppressing angiogenesis for reduced tumor blood supply and growth	Jiang et al. 2022; Chen et al. 2017; Diao et al. 2020)
Esophageal squamous cancer	Constitutive activation of NF-κB, promoting lymph node metastasis	Zhu et al. 2019; Zhao et al. 2022)
Lung cancer	Inhibiting NF-κB activation for diminishing the proliferation and invasion	Zhan et al. 2015; Altinoz et al. 2016; Choi et al. 2014)

## Conclusion

Targeted TRIM3 therapy has certain possibilities and has become one of the research hotspots in the field of cancer therapy. Future, by targeting overexpressed TRIM3 in individual tumors, a personalized treatment regimen can be achieved to improve cure and survival rates. In addition, compared with traditional chemoradiotherapy and other therapies, TRIM3-targeted therapies may have better specificity and reduce damage to normal cells and side effects. However, the current targeted therapy for TRIM3 is still in the early stages, and there are still some challenges and limitations: Similar to other targeted therapies, TRIM3-targeted therapy may also face resistance problems.

In summary, TRIM3 is an ubiquitin-related protein, which is related to cell proliferation, invasion, metastasis, cell cycle, apoptosis, etc., during the occurrence and development of tumors. However, due to the heterogeneity and complexity of tumors, the functional mechanism of TRIM3 in tumors is not fully understood, and its specific signaling pathway and molecular mechanism need to be further clarified. This makes it a challenge for us to use TRIM3 molecules to intervene in tumor development. In future, we will further explore the functional mechanism of TRIM3 molecules in different types of tumors and explore the expression patterns and functional changes of TRIM3 in different tumor subtypes, which may provide valuable insights for molecular diagnosis and gene therapy of cancer patients at the gene level in future and have important clinical significance for diagnosis and treatment.

In my opinion, first of all, TRIM3 is important in tumor diagnosis. Examining the expression levels of TRIM3 in different types of tumors can assist physicians in determining the type and grade of the tumor and predicting the prognosis of the patient. This provides an important reference for clinicians and helps to develop more individualized and precise treatment plans. The study of TRIM3 antitumor mechanism also has a positive impact on the development of treatment guidelines. The in-depth exploration of the relationship between TRIM3 and tumor development can provide a basis for the formulation of more scientific and rational treatment guidelines. For example, in some specific types of tumors, the expression of TRIM3 is closely related to the sensitivity of a certain treatment, so TRIM3 can be used as a predictive indicator to choose the best treatment for patients. The study of TRIM3 antitumor mechanism is also important for evaluating the effectiveness of the treatment methods. By observing the changes of TRIM3 in the course of treatment, it is possible to understand the effect of treatment in time and adjust the treatment program. This helps to improve the accuracy and effectiveness of tumor therapy and reduce unnecessary therapeutic risks. In addition, from the economic point of view, the study of TRIM3 antitumor mechanism also possesses certain value. By gaining a deeper understanding of the relationship between TRIM3 and tumor development, drug use strategies applicable to patients with different types of tumors can be more precisely determined. This can help avoid unnecessary drug waste and side effects and improve the efficiency of medical resource utilization.

## Data Availability

The authors confirm that all data generated or analyzed during this study are included in this article.
